# Sorption from Solution: A Statistical Thermodynamic Fluctuation Theory

**DOI:** 10.1021/acs.langmuir.3c00804

**Published:** 2023-09-08

**Authors:** Seishi Shimizu, Nobuyuki Matubayasi

**Affiliations:** † York Structural Biology Laboratory, Department of Chemistry, University of York, Heslington, York YO10 5DD, United Kingdom; ‡ Division of Chemical Engineering, Graduate School of Engineering Science, Osaka University, Toyonaka, Osaka 560-8531, Japan

## Abstract

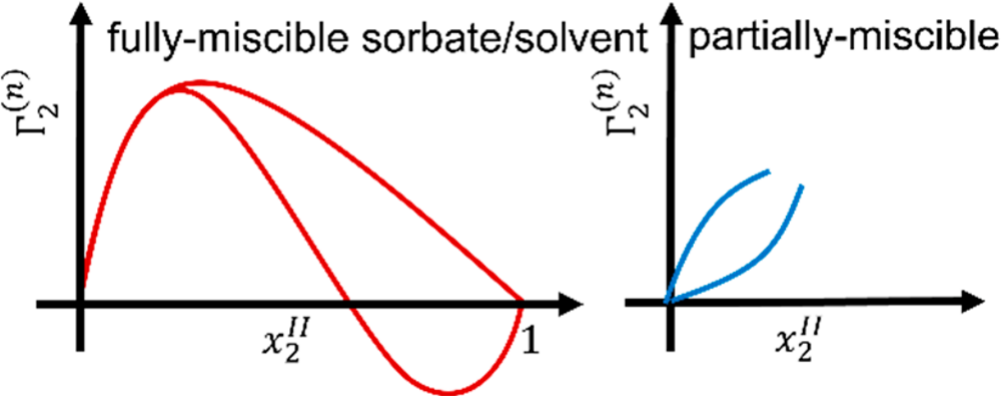

Given an experimental solid/solution sorption isotherm, how can we gain insight into the underlying sorption mechanism on a molecular basis? Classifying sorption isotherms, for both completely and partially miscible solvent/sorbate systems, has been useful, yet the molecular foundation of these classifications remains speculative. Isotherm models, developed predominantly for solid/gas sorption, have been adapted to solid/solution isotherms, yet how their parameters should be interpreted physically has long remained ambiguous. To overcome the inconclusiveness, we establish in this paper a universal theory that can be used for interpreting and modeling solid/solution sorption. This novel theory shares the same theoretical foundation (i.e., the statistical thermodynamic fluctuation theory) not only with solid/gas sorption but also with solvation in liquid solutions and solution nonidealities. The key is the Kirkwood-Buff χ parameter, which quantifies the net self-interaction (i.e., solvent–solvent and sorbate–sorbate interactions minus solvent–sorbate interaction) via the Kirkwood-Buff integral in the same manner as the solvation theory and, unlike the Flory χ, is not limited to the lattice model. We will demonstrate that the Kirkwood-Buff χ is the key not only to isotherm classification but also to generalizing our recent statistical thermodynamic gas (vapor) isotherm, which is capable of fitting most of the solid/solution isotherm types.

## Introduction

Sorption of sorbates (solutes) from solution onto a solid is a fundamental process with many applications (such as contaminant removal), leading to a wealth of solid/solution isotherm data reported so far.^1−3^ However, understanding the underlying molecular interactions, despite its long history,^1−3^ has not been resolved with clarity. Such a difficulty, as will be reviewed below in detail, is caused by the conventional, long-standing approach of adapting vapor (gas) sorption isotherm models for solution isotherms.^4−6^ Our three-fold goal in the present paper is to resolve this historical difficulty:

A. to establish isotherm equations for analyzing sorption from solution based directly on the principles of statistical thermodynamics,
B. to determine the interactions underlying sorption via (A), and
C. to reveal the difference in molecular interactions behind the conventional classifications^7−9^ of solid/solution isotherms.

Our goal, therefore, is to derive analytical isotherm equations for sorption from solution with a clear physical meaning as an alternative to the conventional approach of adapting vapor (gas) isotherms. In addition, achieving these goals will establish the common theoretical foundation that encompasses sorption from solution, sorption of gases and vapors,^10−12^ and solvation in solutions.^13−15^ All three classes of phenomena will then be modeled using the universal language of particle number correlations founded on the statistical thermodynamic fluctuation theory.^16−22^ This universality can be achieved by extending our recent work on the sorption of gases and vapors^10−12^ to solid/solution isotherms. In the following, we will show why this novel, universal approach is indispensable in overcoming the conundrums of conventional approaches.

### Difficulties in Modeling Isotherms

Here we summarize the long-standing difficulties of the conventional isotherm models,^4−6^ mostly being an adaptation of solid/vapor isotherms,^4−6^ in modeling solid/solution systems. The conventional models can be categorized as physical, semiempirical, and empirical.^2,3^ The physical models are founded on an assumed sorption mechanism, such as the adsorption sites, layers, and binding constants. The Langmuir model, based on independent, site-specific gas (vapor) adsorption on a uniform surface,^23^ is one of the most commonly used isotherms for solid/solution sorption,^4−6^ more frequently encountered than the Brunauer–Emmett–Teller (BET)^24,25^ and Guggenheim–Anderson–de Boer (GAB),^26−28^ interpreted as evidence that “[m]ultilayer formation is less common in solution than in the gas phase”.^3^ The semiempirical models are usually founded on connecting a plausible physical quantity (e.g., Polanyi’s adsorption potential,^29−31^) to an isotherm with an empirical equation. Examples include the Dubinin–Radushkevich^32−35^ model that has been applied recently to solid/solution isotherms.^36,37^ The empirical models have been proposed without an assumption on the sorption mechanism on a molecular scale and cannot, in principle, be used for interpreting isotherms on a molecular scale.^11,38^ (Yet their physical meaning may be investigated in later studies, such as the attempts to attribute a physical meaning to the Freundlich model.^39,40^) Consequently, our focus is on the physical models, yet adapting gas (vapor) isotherm models for solutions has not been straightforward. Even for the simplest Langmuir model, thermodynamic quantities for adsorption depend on the standard states adopted, leading to widely discrepant interpretations, as has been demonstrated recently.^41−45^ The question, therefore, is not how the gas-phase isotherm model should be adapted for solutions. A clarification is indispensable at a fundamental level of how isotherms for the sorption from solution must be formulated.

### Difficulties in Interpreting Isotherms

Here we summarize another long-standing problem: the lack of conclusiveness of the conventional isotherm models in revealing the underlying sorption mechanism. The standard experimental measure for adsorption from solution is the reduced surface excess.^9^ Unlike gas (vapor) sorption quantified by the amount of sorption, the surface excess signifies the amount of sorbate relative to that of solvent,^2,46^ necessitated by the competitive interface–sorbate and interface–solvent interactions. This key difference has inspired the following three major approaches to gaining an insight into the mechanism underlying a sorption isotherm: (i) separating an isotherm (i.e., relative surface excess) into individual isotherms,^1,2,9^ (ii) evaluating surface/solution partition coefficients,^1,2,9^ (iii) interpreting the constants obtained by fitting isotherm models to experimental data.^1,2,9^ However, the difficulties faced by all these approaches have been recognized. (i) and (ii) involve a number of assumptions on interfacial layers. Our focus is approach (iii), whose problem is three-fold. First, the highly idealized nature of the model may not reflect the reality of the system. For example, a successful fitting of the Langmuir model to a heterogeneous porous sorbent does not prove the formation of a monolayer with a uniform site-specific binding constant, as has been well recognized.^11,46−48^ Second, comparatively successful fitting achieved by several models, each assuming a different sorption mechanism, leads to a multiplicity of interpretations.^10,11,47,49−52^ Currently, there is no principle other than the goodness of fit (e.g., *R*^2^ values) to identify the right isotherm over others.^49^ Third, site-specific binding models are too simplistic to capture surface excess, which has been recognized in the analogous question in biomolecular solvation.^13,14,53−55^ Thus, a clear theoretical guideline is still lacking for the interpretation of experimental isotherms on a mechanistic level.

### Difficulties in Classifying Isotherms

**Figure 1:**
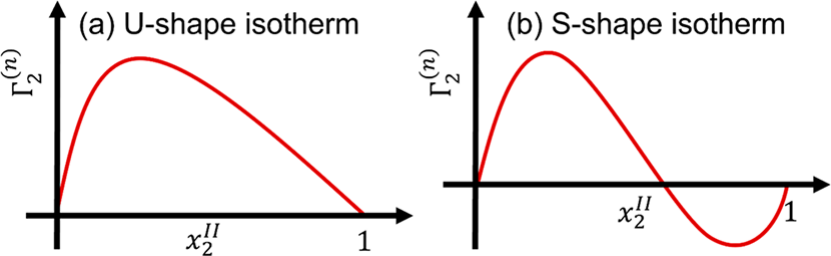
IUPAC classification (1986)^9^ of the sorption isotherms from the completely miscible systems of solvent (species 1) and sorbate (species 2), in which the reduced surface excess, Γ_2_^(*n*)^, is sketched against the mole fraction *x*_2_^II^ of the sorbate in the solution phase (denoted as reference system II in the Theory section). The older classification by Nagy and Schay^61^ provides three further subcategories for the U-shape and two for the S-shape.

**Figure 2:**
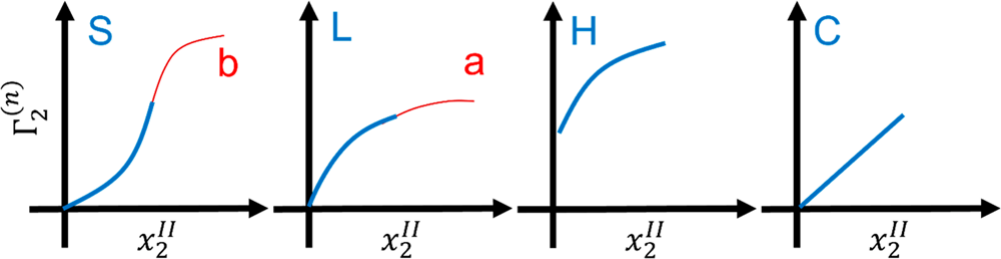
Four main classes of isotherms from partially miscible solvent (species 1) and sorbate (species 2) proposed by Giles et al.,^8^ termed S, L (“Langmuir”), H (“high affinity”), and C (“constant partition”). The IUPAC report (1986) identifies “the two extreme forms”^9^ a and b (drawn in red) as the further continuation of the initial slopes by Giles et al.

The lack of clarity, arising from the conventional approaches (i.e., adapting vapor isotherm models to solutions), leads to ambiguity in the mechanistic basis for classifying isotherms. In contrast to solid/vapor systems, for which the IUPAC classification into six isotherm types has been well established,^56−59^ several approaches are concurrent for solid/solution isotherms for each of the “completely miscible” and “partially miscible” solution phase behaviors.^4,9,60^ For completely miscible systems, IUPAC (1986) has identified the two major classes: the inverted U-shape and the S-shape isotherms (Figure 1).^9^ The two shapes each are divided further into three and two subshapes, respectively, by an earlier system by Nagy and Schay.^61^ For partially miscible systems, there are four main classes of isotherms according to Giles et al.:^4,8,60^ S, L (“Langmuir”), H (“high affinity”), and C (“constant partition”), which are distinguished from one another “by their initial slope”^8^ (Figure 2). The meaning of the “initial slope” was later clarified as the isotherm’s second-order derivative.^60^ Of these classes, the IUPAC report (1986) has identified Classes S and L with saturation as “the two extreme forms”^9^ (Figure 2). Even though the possible molecular mechanisms behind the classifications have been speculated,^8,61^ such discussions suffer from the same set of limitations on the isotherm models and their interpretations summarized in the previous two subsections.

### Need for Statistical Thermodynamics

Here we propose what needs to be done to overcome the conundrums over modeling, interpreting, and classifying solid/solution isotherms, as summarized above. First, surface excess must be understood in terms of the interface–sorbent and interface–sorbate distribution functions, following the state-of-the-art in statistical thermodynamics^62,63^ (rather than the classical site-specific binding approaches^26,64−67^) in order to overcome the difficulties caused by the current isotherm models. Second, isotherm equations must be expressed analytically and, at the same time, founded on the distribution functions. To fulfill these conditions, our recent achievements in the two adjacent areas will be synthesized. The first is the universal theory of solid/vapor isotherms with a clearer physical interpretation of their parameters.^10,11,47,50−52^ This has been achieved by adopting the net (integrated) molecular distributions (i.e., the Kirkwood-Buff integrals and the excess numbers) as the measure of interactions.^10,11,47,50−52^ The second is the statistical thermodynamic reformulation of the preferential solvation theory^68,69^ (founded also on the Kirkwood-Buff integrals^13,14,70^) and its mathematical analogy to the Gibbs isotherm^68,69^ (whose precise nature had been a subject of controversy^53−55^). In both achievements, a direct determination of the net (integrated) distribution functions from experimental data has been demonstrated to be crucial for clarifying the underlying molecular mechanism, especially when the complexity of the system prevents us from building an accurate molecular-based model or when several models in competition lead to controversies.^15,47^

### Scope

Our goal is to develop isotherm equations for sorption from solution with a clear microscopic interpretation to overcome the difficulties caused by the adaptation of gas (vapor) isotherm models. The objectives of this paper are

(A) to establish a general and rigorous statistical thermodynamic foundation for solid/solution isotherms;
(B) to derive statistical thermodynamic isotherms with a clear microscopic interpretation via the mono-, di-, and trisorbate interaction parameters at the interface;
(C) to clarify the statistical thermodynamic foundation for the classifications of solid/solution isotherms.

The theory developed in (A) will provide the universal interpretation principle of an isotherm based on the relationship between its gradient and the interface-solution concentration fluctuation difference. In (B), we will focus on extending one of the two types of isotherm equations identified in our previous work on solid/vapor sorption (i.e., the “ABC isotherm” for IUPAC Types I–III^11,47,48^) to solid/solution systems. The statistical thermodynamic general principle (A), assisted by the ABC isotherm (B), reduces the isotherm classes into a single parameter in (C).

## Theory

### Setup

We consider a solid–liquid interface of arbitrary interfacial shape, ruggedness, and porosity.^10^ We denote the “surface” by *s* while the molecular component that comprises the surface by *e* (*e* for sorb*e*nt). The solid surface faces the solution phase consisting of solvent (species 1) and sorbate (species 2). The only postulate that we will introduce is the finite-ranged nature of the interface.

### Strategy

Our goal is to derive analytical isotherm equations whose parameters have a clear interpretation that can be achieved through a connection to the net (integrated) molecular distribution functions. The number fluctuations and molecular distribution functions are most clearly related via the grand canonical partition function^16^ to the gradient of an experimental isotherm. In addition, our novel approach has the following additional features: (i) the capacity to deal with arbitrary interfacial geometries (via the generalized Gibbs isotherm in the Theoretical Foundation subsection) and (ii) an efficient calculation technique to handle ensemble transformations (statistical variable transformation in the Universal Measures of Interactions subsection).

### Scope

Analytical isotherm equations derived in this paper will be founded on the generalized Gibbs isotherm, assume the finite-ranged nature of the interface, and adopt the expansion via mono-, di-, and trisorbate interactions at the interface of arbitrary geometry. Such an approach cannot be applied to (i) sigmoidal isotherms arising from a cooperative sorption of many sorbate molecules^50^ and (ii) highly heterogeneous surfaces^51^ that require the consideration of statistically independent microscopic patches. Generalizing our theory to (i) and (ii), already achieved for gas (vapor) isotherms,^50,51^ will be carried out in a forthcoming paper. Note that the mono-, di-, and trisorbate interactions will be captured via spatially integrated distribution functions, which, on the one hand, facilitates the derivation of an analytical isotherm equation yet, on the other hand, demands statistical sampling of sorbate distributions when comparing with atomistic simulation.

### Theoretical Foundation

#### The Generalized Gibbs Isotherm

**Figure 3:**
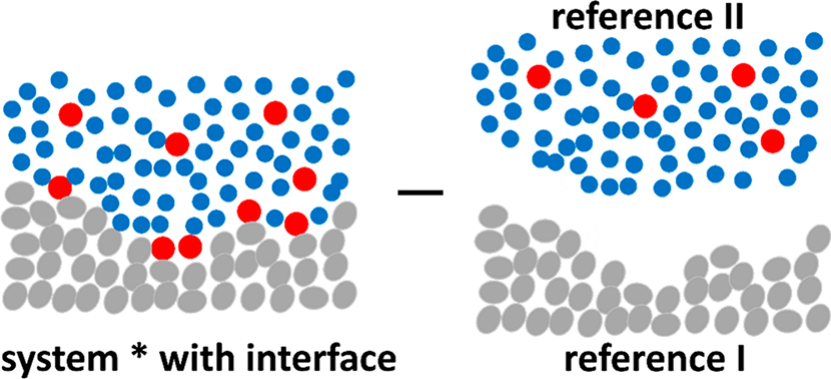
A schematic representation of the interfacial effect as the difference between the system with an interface (left) and the references systems I and II. The sorbent (species *e*), solvent (species 1), and sorbate (species 2) molecules are denoted schematically by gray, blue, and red, respectively. (Note that there is no restrictions on the molecular size and shape).

Following Gibbs,^71^ we consider an interface as the difference between the system (denoted by *, containing the interface) and the two reference systems, that are free of the interfacial effect, on the solid side (denoted by *I*) and the solution side (denoted by *II*), as illustrated schematically by Figure 3.^72^ Here we take a statistical thermodynamic approach based on ensembles and Legendre transforms,^10,11,50^ instead of a thermodynamic approach based on a trio of the Gibbs–Duhem equations.^72^ The thermodynamic functions for the grand canonical ensembles (Ω) are expressed for the system that contains an interface (denoted by the superscript *) and the reference systems *I* and *II*, as
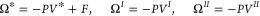
1where *F* is the interfacial free energy, *V* is the volume, and the pressures (*P*) of the system and the reference states are set as identical.^72^ Using Legendre transform, we construct the partially open ensembles, closed to species *e* but open to species 1 and 2, whose thermodynamic potentials (Y*, *Y*^*I*^, and *Y*^*II*^) are expressed as
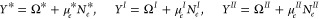
2where μ is the chemical potential and the species have been denoted by the subscript. Under phase equilibrium (μ_e_^*^ = μ_*e*_^*I*^ = μ_*e*_^*II*^), eqs 1 and 2 can be combined to yield
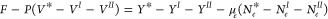
3Following Gibbs, we employ the volume conservation condition (V* = *V*^*I*^ + *V*^*II*^).^72^ Moreover, we impose *N*_e_^*^ = *N*_*e*_^*I*^ + *N*_*e*_^*II*^ for sorbent, which is equivalent to introducing the Gibbs dividing surface. (Note that the interfacial coordinate system, required to define the concentration profile in the conventional introduction of the dividing surface, is unnecessary; our approach, therefore, can handle rugged and porous interfaces for which coordinate systems are difficult to define.^10^) Consequently, eq 3 is simplified as
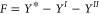
4Thus, the solid-solution interfacial free energy *F* has been written in terms of the partially open ensembles as a generalization of our previous papers on solid–vapor interface.^10,11,47,50−52^

#### Surface Excess for Arbitrary Interfacial Geometry

The surface excess of sorbate (⟨*N*_2_^*^⟩ – ⟨*N*_2_^*I*^⟩ – ⟨*N*_2_^*II*^⟩) and solvent (⟨*N*_1_^*^⟩ – ⟨*N*_1_^*I*^⟩ – ⟨*N*_1_^*II*^⟩) results from the μ_2_-derivative of the interfacial free energy in the {*T*, *V*, *N*_*e*_, μ_1_, μ_2_} ensemble, as
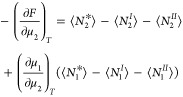
5awhere *T* is the temperature and ⟨ ⟩ expresses ensemble averaging. In deriving eq 5a, we have used the semigrand partition function for the {*T*, *V*, *N*_*e*_, μ_1_, μ_2_} ensemble (i.e., open to species 1 and 2 but closed to species *e*). The ensemble-based derivation of eq 5a is a straightforward extension of the one for gas (vapor) sorption presented in section 2 of ref (10). Here we introduce the following two postulates. The first is that the interface is finite ranged; hence, the interfacial subsystem, thick enough to contain the interface, contains all the interfacial effects. This means that there is no difference in the distribution of sorbent between the system and the reference systems outside the region covered by the interfacial subsystem. From now onward, we use lower-case symbols to denote the numbers (*n*_*i*_) within the interfacial subsystem with volume (*v*). Using this notation, the generalized Gibbs dividing surface condition pertains to the number of sorbent molecules in the interfacial subsystem (*n*_e_^*^) and those in the reference systems that cover the same volume (*n*_*e*_^*I*^ and *n*_*e*_^*II*^), which can be expressed as *n*_e_^*^ = *n_e_^I^* +*n*_*e*_^*II*^. Second, in evaluating  in eq 5a, we postulate that neither species 1 nor 2 penetrate the solid surface (i.e., ⟨*n*_2_^*I*^⟩ = ⟨*n*_1_^*I*^⟩ = 0). Consequently, using the Gibbs–Duhem equations for the reference system *II*, we obtain
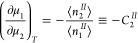
5bwhere *C*_2_^*II*^ = ⟨*n*_2_^*II*^⟩/⟨*n*_1_^*II*^⟩ represents the mole ratio in the reference system *II* (solution). Under the two postulates, eq 5a can be simplified as
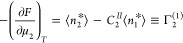
6aHere, the right-hand side of eq 6a, denoted by IUPAC as Γ_2_^(1)^, is referred to as the “relative surface excess of 2 with respect to 1”.^9^ Experimentally, Γ_2_^(*n*)^ = *x*_1_Γ_2_^(1)^, the reduced surface excess, is directly accessible.^9^ Note that Γ_2_^(1)^ can be made intensive when it is divided by the sorbent mass, which is in line with the common practice of reporting Γ_2_^(*n*)^.^73,74^ It is useful to express Γ_2_^(1)^ in terms of the solvent–surface and sorbate–surface Kirkwood-Buff integrals, *G*_*s*1_ and *G*_*s*2_, as
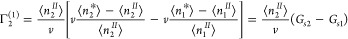
6bFor solid/gas sorption, the absence of the solvent reduces Γ_2_^(1)^ to ⟨*n*_2_^*^⟩ (where ⟨*n_2_^I^*^I^⟩ is negligible); hence, eq 6a is a generalization of our previous theory.^10,11,47^ However, for solid/solution isotherms, the individual solvent and sorbent contributions to Γ_2_^(1)^, according to the IUPAC report, “can only be calculated on the basis of some model of the interfacial region, and have no place in the primary presentation of experimental data”.^9^ Thus, we have introduced the relative surface excess via eq 6a, in a manner valid for any interfacial geometry or porosity.

### Universal Measures of Interactions

#### Quantifying Sorbate Interactions from the Gradient of an Isotherm

A sorption isotherm for the solid-solution interface describes how the relative surface excess Γ_2_^(1)^ depends on the sorbate concentration. Our goal is to elucidate the underlying sorption mechanism from an isotherm. Recently, we have shown that the gradient of a solid–vapor sorption isotherm reveals the strength of sorbate–sorbate interaction whose quantitative measures are the Kirkwood-Buff integral and excess number which are both net (integrated) distribution functions.^10,11,47^ Here we generalize it to adsorption from solution. This involves differentiation of the surface excess Γ_2_^(1)^ (eq 6a) with respect to *a*_2_. Carrying this out directly in our semiopen ensemble (denoted as {*T*, *v*, *n*_*e*_, μ_1_, μ_2_}) incurs cumbersome algebra. However, significant simplification can be achieved by exploiting our new approach to ensemble transformation.^21,22^ Because of the technical nature, the detailed derivation will be presented in the Supporting Information, while we summarize below the main points:

- Γ_2_^(1)^ is invariant under the {*T*, *v*, *n*_*e*_, μ_1_, μ_2_} ↔ {*T*, *v*, *n*_*e*_, *n*_1_, μ_2_} transformation (‘Ensemble invariance of the surface excess’ in the Supporting Information section A).
- *a*_2_-derivatives can be evaluated more easily in the {*T*, *v*, *n*_*e*_, *n*_1_, μ_2_} ensemble (‘Calculating the gradient of surface excess via ensemble independence’ in the Supporting Information section B).
- An ensemble average in {*T*, *v*, *n*_*e*_, μ_1_, μ_2_} (denoted as ⟨ ⟩_{*T*, *v*, *n*_*e*_, μ_1_, μ_2_}_) can be calculated straightforwardly from the one in {*T*, *v*, *n*_*e*_, *n*_1_, μ_2_} (denoted as ⟨ ⟩_{*T*, *v*, *n*_*e*_, *n*_1_, μ_2_}_) via statistical variable transformation (‘Calculating the gradient of surface excess via ensemble independence’ in the Supporting Information section B).^21,22^

The steps of the derivation, because of their technicality, are presented in detail in the Supporting Information. Thus, we derived our fundamental equation in two different representations. The first relates the gradient of an isotherm, , to the difference in concentration fluctuation between the interface (*) and solution reference state (*II*), via
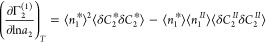
7aThe second representation, equivalent to eq 7a, can be expressed in terms of the number fluctuations via
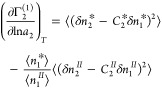
7bThus, we successfully established a link (via eq 7a or 7b) between the gradient of an isotherm and the underlying fluctuations. The solid/solution relationship (eq 7b) contains the solid/gas counterpart as its special case when the solvent is dilute and the reference state is negligible (Supporting Information section B). However, the fundamental relationships (eqs 7a and 7b) are not practical to apply. They must be expressed in terms of the net (integrated) distribution functions so that there is a clear link to the interactions between the molecular species involved. This will be achieved in the next paragraph.

#### The Interfacial Kirkwood-Buff χ Parameter

Our goal has been to elucidate the sorption mechanism from the gradient of an isotherm. The key to achieving this goal is the relationship between the concentration fluctuations (eqs 7a and 7b) and the Kirkwood-Buff integrals (*G*_*ij*_, between the species *i* and *j*, see Supporting Information section C for derivation) via
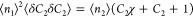
8a
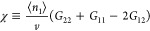
8bwhere we have introduced the *Kirkwood-Buff χ* parameter via eq 8b, which will be used for * and *II*. Note the involvement of sorbate–sorbate (*G*_22_), sorbate–solvent (*G*_12_), and solvent–solvent (*G*_11_) Kirkwood-Buff integrals in eq 8b, as compared to gas (vapor) sorption for which only *G*_22_ is present.^11,48^ What is crucial for a molecular-based interpretation is the relationship between *G*_*ij*_ and the distribution function between the species *i* and *j*, *g*_*ij*_(***r***) with their relative configuration ***r***, via^11^
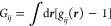
8cThe term, i.e., “the Kirkwood-Buff χ parameter”, has been inspired by its relationship to the activity coefficient, γ_1_, in dilute binary solutions,^75^
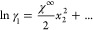
9where *x*_2_ is the mole-fraction of species 2 and χ^∞^ is the limiting value at *x*_2_ → 0; eq 9 is analogous to the role of the Flory–Huggins χ parameter,^76^*χ*_*FH*_, present in the following equation:
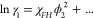
10a
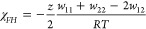
10bwhere ϕ_2_ is the volume fraction of species 2, *z* is the number of contacts, and *w*_*ij*_ is the contact energy between species *i* and *j*, yet, in practice, the mole fraction *x*_2_ is widely used in place of ϕ_2_.^76^ (Note that we have not incorporated the factor 1/2, that are present in both the Kirkwood-Buff and Flory–Huggins theories, into the definition of χ in eq 8b simply to keep our subsequent equations simpler.) According to the Kirkwood-Buff theory, the more positive *G*_*ij*_ is, the more attractive the interaction is between species *i* and *j*. In the Flory–Huggins theory, attractive interaction comes with a negative contact energy, *w*_*ij*_, which justifies the negative sign in eq 10b. Both χ parameters are a measure of self-interaction compared to the mutual. Consequently, adopting the Kirkwood-Buff χ parameter as the measure of interaction, we can express the isotherm gradient (Supporting Information section C), as
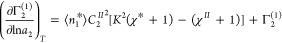
11where *K* = *C*_2_^*^/*C*_2_^*II*^ signifies the sorbate–solvent exchange constant between the interface and solution phase. Equation 11 involves the Kirkwood-Buff χ parameters for the interface (∗) and solution (*II*). Equation 11 will be converted into a simple, usable form in the next subsection.

### Modeling and Classifying Isotherms

#### The Activity-Based ABC Isotherm

**Figure 4:**
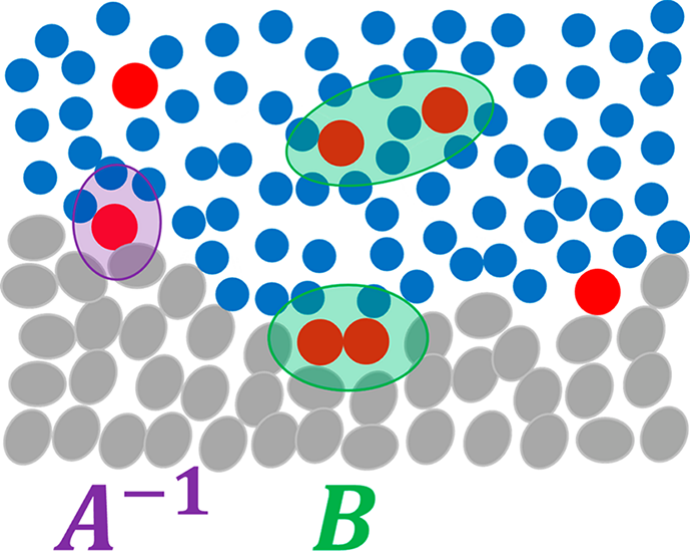
A schematic representation of the isotherm parameters, *A* and *B*. *A*^–1^ (purple) signifies the preferential sorbate–surface (over solvent–surface). *B* (green) signifies the surface–bulk difference of self-interaction (sorbate–sorbate and solvent–solvent over sorbate–solvent. The parameter *C*, which will not be the main focus of this paper, involves triplet correlations.

Even though we were able to express the gradient of an isotherm in terms of the interfacial and solution χ parameters, eq 11 is still too complicated for interpretation. The goal of this subsection is two-fold: (i) to identify a simpler measure determinable from an isotherm and (ii) to derive an isotherm equation to analyze experimental data. To achieve our two-fold goal, here we generalize our general ABC isotherm for gases and vapors to the adsorption from solution. After some algebra (‘The ABC isotherm for solutions’ in the Supporting Information section D), eq 11 can be rewritten concisely as
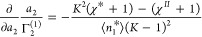
12Following our recent work,^11,47^ we introduce the following activity expansion
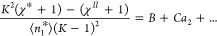
13and integrating eq 12 together with eq 13 yields the following isotherm equation (Supporting Information section D):
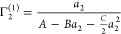
14awhere *A*, via
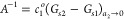
14bsignifies the sorbate–surface preferential interaction over solvent–surface (Figure 4), and *B*, via
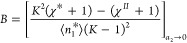
14csignifies the surface-solution χ difference (Figure 4). Note that *c_1_^o^* is the bulk molar concentration of the solvent. Here, *K*^2^ multiplied to χ* + 1 signifies the two sorbate molecules sorbed at the interface in exchange with two solvent molecules; *K* can also be related to *G*_*s*2_ – *G*_*s*1_ (see Supporting Information section D). Note that 1 is present in χ + 1 for both the interface * and the reference solution system *II*. Its significance can be elucidated by considering the case in which species 2 interacts weakly with itself as well as with species 1, such that *G*_22_ ≃ 0 and *G*_12_ ≃ 0. In this case, χ ≃ −1 because *G*_11_ ≃ −*v*/⟨*n*_1_⟩ for a pure solvent.^14^ Consequently, a noninteracting sorbate gives χ + 1 ≃ 0 which serves as the baseline. The parameter *C* is more complicated, involving triplet interactions, yet it will be shown to play a minor role in elucidating the difference in molecular interactions between different classes of isotherms. Our solid/solution theory can be shown to be a generalization of our previous solid/gas theory^10,11^ (Supporting Information section D). Just like its solid/gas counterpart, the solid/solution ABC isotherm (eq 14a) is based on the sorbate activity expansion (eq 13), which will be unsuitable when multiple sorbate molecules sorb cooperatively.^50,51^ Moreover, for heterogeneous surfaces, multiple isotherm terms may become necessary for accurately capturing the isotherm.^48,51^

#### The Mole-Fraction-Based ABC Isotherm

We derived our theory, including the ABC isotherm for solutions, using sorbate activity as the concentration scale. However, it is common to use mole fraction as the measure of sorbate concentration in reporting isotherms.^9^ Consequently, we need to present our ABC isotherm using the mole fraction in the solution, *x*_2_^*II*^. The derivation (whose details are found in ‘The ABC isotherm in mole-fraction scale’ in the Supporting Information section E) is facilitated by the fact that the isotherm parameters (*A*, *B*, *C*) are evaluated at the dilute concentration limit, which leaves the parameters *A* and *B* unchanged from the activity-based isotherm (eq 14a), as
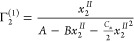
15where *C*_*x*_ is different from *C* in eq 14a due to the contributions from the activity coefficient involved in the first-order term in *a*_2_, yet is unimportant when elucidating the isotherm types. Thus, the ABC isotherm, through its application to isotherm fitting, enables the quantification of interactions underlying an isotherm via *A* and *B*.

#### The Cubic Isotherm

Unlike sorption from the gas phase, Γ_2_^(1)^ from solution may become negative, for which the ABC isotherm is not suitable (because its denominator diverges at Γ_2_^(1)^ = 0). To circumvent this problem, we can derive the cubic isotherm as an alternative isotherm equation (‘The cubic isotherm’ in the Supporting Information section F), as
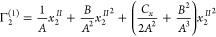
16Since the cubic isotherm (eq 16) has been derived from the same fundamental relationship (i.e., eq 12 = eq D2, rewritten as eq F1 in Supporting Information section F) as the ABC, the same set of parameters (*A*, *B*, *C*_*x*_) as the ABC isotherm is determinable by analyzing the cubic isotherm. It should be noted that a negative Γ_2_^(1)^ signifies the repulsion of species 2 (sorbate) from the interface, when measured relative to species 1 (solvent). More simply, Γ_2_^(1)^ is negative when the interface is more favorable to the solvent than to the sorbate.

## Results and Discussion

### Modeling Isotherms

**Figure 5:**
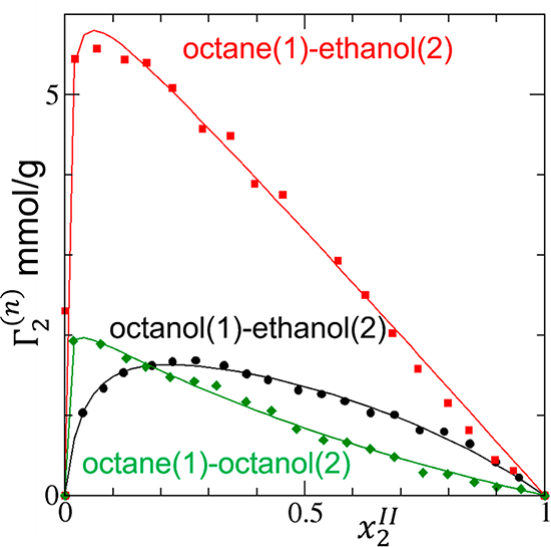
Modeling the sorption isotherm (surface excess Γ_2_^(*n*)^ against the mole fraction *x*_2_^*II*^ of sorbates) on SBA-16 silica sample of the following (solvent(1)/sorbate(2)) combinations: *n*-octane/ethanol (red), octanol/ethanol (black), and *n*-octane/octanol (green). The ABC isotherm (eq 17) was used to fit the experimental data, measured by Rockmann and Kalies,^73^ with the resultant parameters summarized in Table 1.

**Figure 6:**
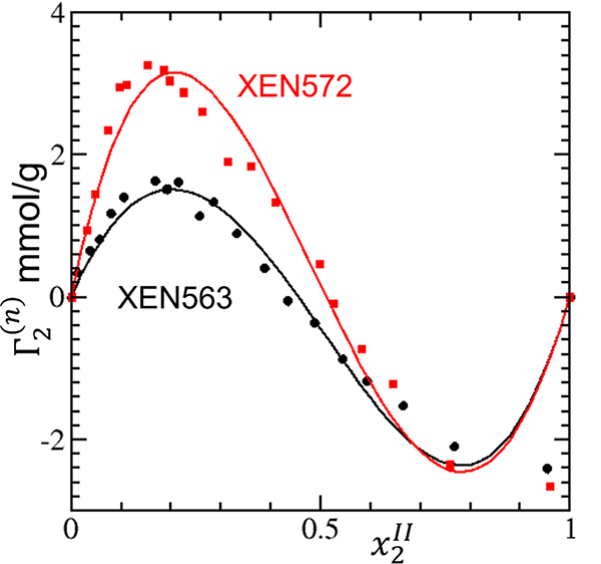
Modeling the sorption isotherm (surface excess Γ_2_^(*n*)^ against the mole fraction *x*_2_^*II*^ of sorbates) of ethanol/*n*-octane on the carbonaceous Ambersorb adsorbents XEN563 and XEN572. The cubic isotherm (eq 18) was used to fit the experimental data, measured by Kalies et al.,^74^ with the resultant parameters summarized in Table 1.

**Figure 7:**
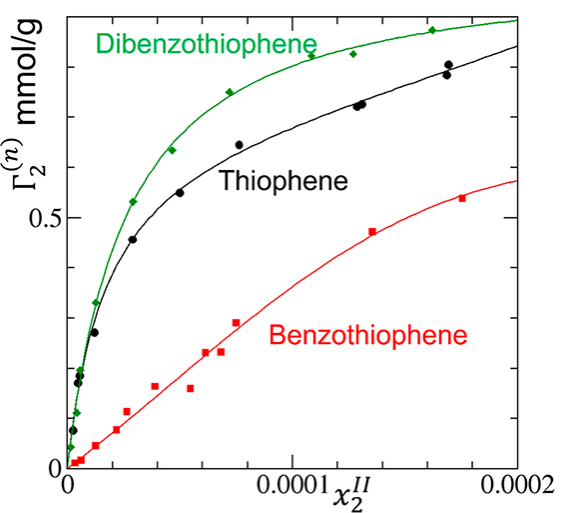
Modeling the sorption isotherm (surface excess Γ_2_^(*n*)^ against the mole fraction *x*_2_^*II*^ of sorbates) of thiophene (black circles), benzothiophene (red squares), and dibenzothiophene (green diamonds) in water on the Cu-BTC metal–organic framework measured by Liu et al.^77^ The ABC isotherm (eq 17) was used to fit the experimental data with the resultant parameters summarized in Table 1.

Given an experimental isotherm, how can we elucidate the underlying mechanism of adsorption from solution? The first step is to fit the isotherm and determine the interaction parameters. In the Theory section, we have provided the following two isotherms (eqs 15 and 16):
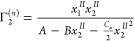
17
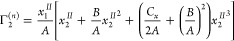
18The Γ_2_^(1)^-based expressions have been converted to the reduced surface excess, Γ_2_^(*n*)^, via Γ_2_^(*n*)^ = *x*_1_Γ_2_^(1)^, which is the quantity directly accessible to experimental measurements.^9^ The ABC isotherm (eq 17) is a model-free generalization of the Langmuir, BET, and GAB models. Note that the same set of parameters (*A*, *B*, *C*_*x*_) results from the two isotherm equations (eqs 17 and 18). While the cubic equation can fit the isotherm when Γ_2_^(*n*)^ changes its sign to become negative, the ABC isotherm, which cannot handle the change in sign, is more suitable for reproducing the isotherm functional shape at low *x*_2_. For the completely miscible solvent–sorbate systems, the ABC isotherm (eq 17) was successful to fit the literature sorption data on SBA-16 silica^73^ (Figure 5) and the cubic isotherm (eq 18) could reproduce the overall shape of the literature sorption data on carbonaceous Ambersorb adsorbents (Figure 6).^74^ The ABC isotherm has been applied also to the partially miscible systems, fitting successfully the literature sorption data on the adsorption of thiophenes on a metal–organic framework (Figure 7).^77^ The parameters determined from fitting are summarized in Table 1. Our approach is advantageous because the same set of parameters (*A*, *B*, and *C*_*x*_) can be determined from the different isotherm equations (eqs 17 and 18).

**Table 1:** Fitting Parameters for the ABC and Cubic Isotherms for Experimental Adsorption Data from Solutions

sorbent	solvent	sorbate	*A*	*B*	*C*
SBA-16^a^	*n*-octane	ethanol	5.59 × 10^–4^	–1.52 × 10^–1^	8.60 × 10^–3^
SBA-16^a^	octanol	ethanol	1.89 × 10^–2^	–4.41 × 10^–1^	4.41 × 10^–1^
SBA-16^a^	*n*-octane	octanol	8.96 × 10^–4^	–4.54 × 10^–1^	–5.56 × 10^–1^
XEN563^b^	ethanol	*n*-octane	6.26 × 10^–2^	–1.21 × 10^–1^	–5.38 × 10^–1^
XEN572^b^	ethanol	*n*-octane	2.93 × 10^–2^	–6.50 × 10^–2^	–2.57 × 10^–1^
Cu-BTC^c^	Water	thiophene	2.44 × 10^–5^	–1.40 × 10°	3.30 × 10^3^
Cu-BTC^c^	water	benzothiophene	2.93 × 10^–4^	6.07 × 10^–1^	–8.86 × 10^3^
Cu-BTC^c^	water	dibenzothiophene	2.64 × 10^–5^	–9.77 × 10^–1^	–2.09 × 10^2^

a Data from Rockmann and Kalies,^73^ using eq 17, with the units in g/mmol.

b Data from Kalies et al.^74^ using eq 18, with the units in g/mmol.

c Data from Liu et al.^77^ measured between *x*_2_^*II*^ = 0 and 1.8 × 10^–4^, using eq 17, with the units in g/mmol.

### Interpreting Isotherms

The second step toward a mechanistic elucidation of an isotherm is the physical interpretation of the isotherm parameters determined from fitting. Our statistical thermodynamic isotherms (eqs 17 and 18) have the following advantages. (i) Despite the use of two different isotherm equations (eqs 17 and 18) to fit three different classes of isotherms (Figures 5–7), the resulting set of parameters (*A*,*B*,*C*_*x*_) are the same, which facilitates comparison between different isotherms. (ii) These parameters (*A*, *B*, *C*_*x*_) have a direct statistical thermodynamic interpretation; they can be expressed in terms of the Kirkwood-Buff integrals and number correlations. Especially important are *A* and *B*; *A*^–1^ is the preferential sorbate–surface interaction at *x*_2_^*II*^ → 0 (eq 14b), and *B* is the difference in the Kirkwood-Buff χ parameter between the interface and solution (eq 14c). (iii) The solid/solution ABC isotherm is analogous to the gas (vapor) ABC isotherm, and the relationship between the two has been made clear (Supporting Information section D). The gas(vapor) ABC isotherms were demonstrated to be capable of modeling IUPAC Types I, II, and III^11,48^ and were shown to be capable of capturing the so-called monolayer-multilayer mechanism used in surface area determination.^47,48^

In our isotherm modeling via eqs 17 and 18, note that *B* in Table 1, except for the one for benzothiophene on Cu-BTC, is negative. A negative *B* is inevitably driven by χ* + 1 at the interface, which is weaker than *χ*^*II*^ + 1 in the solution phase (eq 14c). Since χ* + 1 signifies net self-association at the interface (eq 8b), sorbates are less self-associated at the interface than in the bulk. While self-association is weaker at the interface, the positive *A* in Table 1 signifies, via eq 14b, that the sorbate–interface interaction is stronger than that of the sorbate–solvent. The accumulation of sorbate molecules at the interface does not make them closer together than in the bulk solution but keeps them away from one another, more so than in the solution phase. The possible mechanism for χ* < *χ*^*II*^ could be a strong, specific sorbate–surface interaction with the interface, which could contribute to keeping the sorbates separated at the site–site distance. Sorbate–sorbate separation contributes negatively to *G*_22_^*^ and hence to χ*. Such a mechanism has been observed for the solid/vapor isotherms yet was not captured by the previous models.^11,47^ (To examine the validity of our consideration above, molecular simulations would be helpful). However, we emphasize that sorbate–sorbate separation, caused by the specific interaction between sorbate and surface, has been implicit in the Langmuir model (which is a restricted case of the ABC isotherm^11,47^),
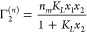
19awhere *n*_*m*_ is the monolayer capacity and *K*_*L*_ is the Langmuir constant. A comparison of eq 19a with the ABC model (eq 17 with *C* = 0) yields the following correspondence:
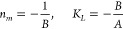
19bA positive Langmuir constant (and a positive monolayer capacity) leads to a negative *B*. A negative *B*, via eq 14c, shows a much smaller interfacial χ* than that of the bulk solution *χ*^*II*^.

We have encountered *B*/*A* when we have shown that sorbate–sorbate exclusion is caused by a site-specific interaction between sorbate and surface.^11,47^ Indeed, *B*/*A* will be demonstrated below to play a central role in classifying sorption isotherms from solutions. For this purpose, we provide its statistical thermodynamic interpretation (Supporting Information, eq D9 section D) as
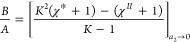
20to which Kirkwood-Buff χ difference between the interface and solution plays a crucial role. (Note that *K*^2^ multiplied to χ accounts for the sorption of two sorbates at the interface, coming from the significance of *B* as representing sorbate pairwise interaction; *K* can also be related to *G*_*s*2_ – *G*_*s*1_ (Supporting Information, eq D10 section D).). Thus, our approach has linked isotherm analysis to χ, which is a universal quantity of interaction in the solution phase instead of relying on overly idealized assumptions to construct multiple isotherm models for separate applications.

### Classifying Isotherms

#### IUPAC (1986) Classification for Completely Miscible Systems

**Figure 8:**
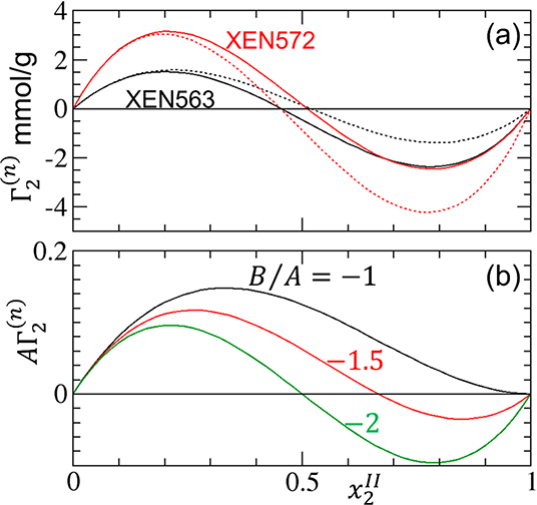
(a) A comparison between the cubic (eq 18, solid lines) and quadratic (dropping the *x*_2_^*II*^(3) term of eq 18, dotted lines) for the fittings of the sorption on carbonaceous adsorbent (Figure 6), showing that the positions, at which the isotherm crosses zero, are determined approximately by the first two terms of the cubic isotherm. (b) The transition from U-shape to S-shape modeled by the normalized quadratic isotherm (eq 18) with the parameters *B*/*A* = −1 (black), −1.5 (red), and −2 (green), showing that *B*/*A* is the key parameter governing the difference between the two shapes.

Two major classes have been identified for completely miscible solvent–sorbate systems: the inverted U-shape and the S-shape isotherms (Figure 1).^9^ Even though we have used the ABC isotherm (eq 17) to fit the U-shape data (Figure 5) and the cubic isotherm (eq 18) for the S-shape data (Figure 6), the same set of parameters (*A*, *B*, and *C*_*x*_) has been determined from the two isotherms. Here we show that *B*/*A* (eq 20) plays a key role in distinguishing the two isotherm shapes. To do so, let us first note that the position at which Γ_2_^(*n*)^ crosses zero does not deviate significantly by neglecting the cubic term in eq 18 (Figure 8(a)). (Note that  is relatively small.) Consequently, *x*_2_^*II*^, at which Γ_2_^(*n*)^ crosses zero, can be evaluated approximately by solving
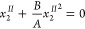
21awhose solution is
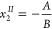
21b

For an isotherm to take an S-shape, , leading to  for the S-shape,  for the U-shape (Table 2). This comparison, when viewed in conjunction with eq 20, has shown that the S-shape reflects a weaker interfacial self-association (relative to the bulk solution) than the U-shape.

**Table 2:** IUPAC S-Shaped and U-Shaped Isotherm Classifications via the Cubic Isotherm Parameters (eq 20)

IUPAC (1986) classification	U-shaped	S-shaped
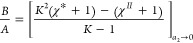	≥ −1	< −1

#### Classification for Partially Miscible Systems

When solvent and sorbates are only partially miscible, there are four main classes of isotherms according to Giles et al.,^4,8^ S, L (“Langmuir”), H (“high affinity”), and C (“constant partition”), that are distinguished from one another “by their initial slope”^8^ (Figure 2), wherein the IUPAC report (1986) identifies Classes S and L with saturation as “the two extreme forms”^9^ (Figure 2). The “initial slope” was later clarified to signify the second-order derivative.^60^ The statistical thermodynamic foundation for this classification can be found by a Maclaurin expansion of eq 17,
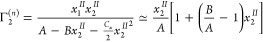
22awherein the classification is reduced to the sign of the second-order derivative, namely, that of . Consequently, Class C, a linear Γ_2_^(*n*)^ = *x*_2_^*II*^/*A*, is realized when . Class S is characterized by a steeper initial slope than linearity, i.e., . Classes L and H exhibit the initial slope less steep than the linearity, hence . These observations are summarized in Table 3. However, the classification can be simplified further^60^ when we plot ln Γ_2_^(1)^ against ln *a*_2_, as
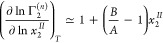
22bwhere the key parameter, *B*/*A*, appears as the gradient (i.e., first-order derivative) of this plot, making the second-order differentiation redundant. To summarize, *B*/*A*, reflecting the interface–solution χ difference, has been demonstrated to play a central role in classifying partially miscible isotherms, as well.

**Table 3:** Classification by Giles et al. via the ABC Isotherm Parameters (eq 17)^a^

Giles et al. classification	S	L	H	C
IUPAC (1986) classification	(b)	(a)	(a)	
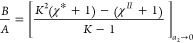	>1	<1	<1	=1

a See Figure 2 for the definitions of classifications.

#### A Statistical Thermodynamic Classification

We have shown that the interface–solution χ difference is the key to the classifications for both the completely miscible and partially miscible systems. For both classifications, *B*/*A*, or the Kirkwood-Buff χ difference (eq 20) plays the key role. However, the *B*/*A* boundaries for the two classifications are at 1 and −1 for partially and completely miscible systems, respectively (Tables 2 and 3). Both classifications are based on Γ_2_^(*n*)^ as the isotherm measure. However, if Γ_2_^(1)^ were to be used as a basis for isotherm classification,
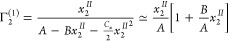
23a
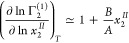
23bthen the second-order derivative of Γ_2_^(1)^ (eq 23a) and the ln-ln gradient  (eq 23b) directly reflect *B*/*A*, namely, the interface–solution χ difference via eq 20. Although *B*/*A* = 1 as the classification boundary for partially miscible systems (Table 3) comes from adopting Γ_2_^(*n*)^ (eq 22a) instead of Γ_2_^(1)^ (eq 23a), *B*/*A* = −1 as the classification boundary for completely miscible systems (Table 2) is common to Γ_2_^(*n*)^ and Γ_2_^(1)^ = Γ_2_^(*n*)^ = 0 between *x*_2_^*II*^ = 0 and 1. Thus, we have clarified the central role of the interface–solution χ difference in the isotherm classification schemes. More detailed classifications (such as by Nagy and Schay^61^ and the subclasses of Giles et al.^8^) will be discussed in a later publication.

### Across Sorption and Solvation

Here we show that our new solution isotherms are valid, even when the solid surface component dissolves into the solution phase. This powerful result stems from the following analogy between the fluctuation solution and the sorption theories. The first is between the surface energy–surface excess relationship (eq 6a) and the preferential solvation theory,
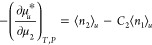
24which relates the μ_2_-dependence on the solvation free energy of a solute (μ_u_^*^) to the preferential solvation ⟨*n*_2_⟩_*u*_ – *C*_2_⟨*n*_1_⟩_*u*_, where ⟨ ⟩_*u*_ signifies the ensemble average in the inhomogeneous ensemble in the presence of a solute molecule and *C*_2_ is the mole ratio in the bulk solution.^68,78^ The second parallel is on the second-order derivatives between the interfacial derivative (eq B5 in Supporting Information),
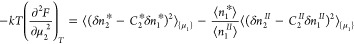
25and the solvation derivative (eq 46 of ref (21) with the indexes 1 and 2 swapped)
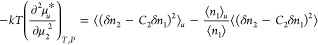
26These parallel relationships demonstrate the solvation-sorption analogy. Indeed, from eqs 24 and 26, it follows that
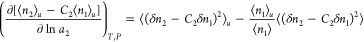
27which is analogous to the fundamental relationship for adsorption from solution (eq 7b). This analogy has a practical significance. When a part of the solid component dissolves into the solution, the surface excess (⟨*n*_2_^*^⟩ – *C*_2_^*II*^⟨*n*_1_^*^⟩) becomes preferential solvation (⟨*n*_2_⟩_*u*_ – *C*_2_⟨*n*_1_⟩_*u*_) (where *C*_2_^*II*^ = *C*_2_) and the interface-solution fluctuation difference 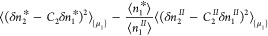
28becomes the solvation-bulk difference 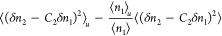
29while retaining the mathematical form. This analogy implies the validity of our solution-phase sorption theory even when some of the solid components dissolve into solution, which will be discussed in a forthcoming paper.

## Conclusions

It has long been customary to analyze solid/solution sorption isotherms using the adapted solid/gas isotherm models, such as Langmuir, Freundlich, or BET. Such an adaptation, however, suffers from a lack of clarity when it comes to interpreting the model parameters derived originally for gas adsorption. We aimed to bring clarity by showing that the underlying sorption mechanism can indeed be obtained by fitting solid/solution isotherms.

The first step toward achieving this aim was to establish a general and rigorous statistical thermodynamic foundation for solid/solution isotherms, starting from the generalized Gibbs isotherm, applicable to any interfacial geometry. On this foundation, we have introduced the Kirkwood-Buff χ parameter as the measure of net self-interaction at the interface and in the solution. Unlike the Flory χ based on the lattice model, the Kirkwood-Buff χ is assumption-free and appears widely in the solution theory, such as in the activity coefficient^75^ and cooperative solubilization by hydrotropes and surfactants.^68,78,79^ Hence the use of Kirkwood-Buff χ establishes a common language between sorption and solution.

On this theoretical foundation, we have derived the two isotherm equations (i.e., ABC and cubic). Both isotherms share the same set of parameters, with a clear microscopic interpretation. The key is the interface–solution difference of the Kirkwood-Buff χ parameter, which can be evaluated by fitting experimental isotherms using the ABC and cubic. The ABC and cubic isotherms share their parameters, yet they exhibit suitability for different classes of isotherms in partially and fully miscible systems. The ABC isotherm, a model-free generalization of Langmuir, BET, and GAB, is useful for the partially miscible systems and the U-shaped isotherms in the fully miscible systems. The cubic isotherm is suitable for S-shaped isotherms in fully miscible systems. The Kirkwood-Buff χ parameter not only provides the key insight into the underlying sorption mechanism but also is the defining signature of isotherm classifications. Moreover, due to its relationship to the molecular distribution functions, Kirkwood-Buff χ can be used to compare an experimental isotherm to a simulation.

Thus, we have established a tractable sorption theory for solid-solution and solid–gas systems, formulated in the universal language of the Kirkwood-Buff χ parameter. At the present stage, our theory cannot be applied to (i) cooperative sorption isotherms^50^ that may not be captured by the mono-, di-, and trisorbate expansions adopted in this paper and (ii) complex isotherms for highly heterogeneous surfaces^51^ that require the consideration of statistically independent microscopic patches that have not been considered in this paper. However, these problems have been resolved for gas (vapor) isotherms.^50,51^ Their generalization to solid/solution isotherms will be carried out in a forthcoming paper.

## Supporting Information Available

Ensemble invariance of the surface excess; Calculating the gradient of surface excess via ensemble independence; The “Kirkwood-Buff χ” parameter; The ABC isotherm for solutions; The ABC isotherm in mole-fraction scale; The cubic isotherm (PDF)
