# Supplementary material for: Sorption from Solution: A Statistical Thermodynamic Fluctuation Theory
Source: Langmuir. 2023 Sep 8;39(37):12987–98. doi: 10.1021/acs.langmuir.3c00804 (PMC10515636; doi:10.1021/acs.langmuir.3c00804)
Supplement: Supplementary file 1 — la3c00804_si_001.pdf [file la3c00804_si_001.pdf]

Supporting information

Sorption from Solution: A Statistical Thermodynamic Fluctuation Theory

Seishi Shimizu[1,*] and Nobuyuki Matubayasi[2]
[1]York Structural Biology Laboratory, Department of Chemistry, University of York, Heslington, York YO10 5DD, United Kingdom.
[2]Division of Chemical Engineering, Graduate School of Engineering Science, Osaka University, Toyonaka, Osaka 560-8531, Japan

Corresponding Author: Seishi Shimizu: York Structural Biology Laboratory, Department of Chemistry, University of York, Heslington, York YO10 5DD, United Kingdom.
Email: seishi.shimizu@york.ac.uk

Table of Contents

A. Ensemble invariance of the surface excess. p.S1
B. Calculating the gradient of surface excess via ensemble independence. p.S2
C. The "Kirkwood-Buff χ" parameter. p.S3
D. The ABC isotherm for solutions. p.S4
E. The ABC isotherm in mole-fraction scale. p.S5
F. The cubic isotherm. p.S6

A. Ensemble invariance of the surface excess

Here we show that surface excess is invariant under ensemble transformation. We start from the partially open ensembles, Y, in eq 4, that are open to species 1 and 2 but are closed to species e, with the Gibbs dividing surface condition for e. Our objective is to carry out a μ_2 differentiation. To facilitate calculation, we introduce Y', an ensemble open to species 2 but closed to species e and 1, as

$$Y'^* = Y^* + \mu_1 N_1, \quad Y'^I = Y^I + \mu_1^I N_1^I, \quad Y'^{II} = Y^{II} + \mu_1^{II} N_1^{II} \qquad \text{(A1)}$$

Combining eq A1 with eq 4, we obtain

$$F = Y'^* - Y'^I - Y'^{II} - \mu_1 (N_1^* - N_1^I - N_1^{II}) \qquad \text{(A2)}$$

Now we carry out the μ_2-derivative of eq A2, as

$$-\left(\frac{\partial F}{\partial \mu_2}\right)_T = \langle n_2^* \rangle_{\{n_1\}} - \langle n_2^I \rangle_{\{n_1^I\}} - \langle n_2^{II} \rangle_{\{n_1^{II}\}} + \left(\frac{\partial \mu_1}{\partial \mu_2}\right)_T (n_1^* - n_1^I - n_1^{II}) \qquad \text{(A3)}$$

where we have emphasized in { } that the mean sorbate numbers have been evaluated in the constant n_1 ensembles. Note that we have incorporated the finite distance nature of the interface, which has been denoted by the lowercase for numbers. From now onwards, for simplicity, we do not consider the distribution of sorbate and solvent molecules inside the solid material e, hence eq A3 can be simplified as

$$-\left(\frac{\partial F}{\partial \mu_2}\right)_T = \langle n_2^* \rangle_{\{n_1\}} - \langle n_2^{II} \rangle_{\{n_1^{II}\}} + \left(\frac{\partial \mu_1}{\partial \mu_2}\right)_T (n_1^* - n_1^{II}) \qquad \text{(A4)}$$

For the evaluation of $\left(\frac{\partial \mu_1}{\partial \mu_2}\right)_T$, we are under the postulate that the component e does not dissolve into the solution and the species 1 and 2 do not penetrate the solid sorbent. This is in agreement with the common practice that the reference system (II) is commonly treated like a bulk

solution. Consequently, we employ the Gibbs-Duhem equation for the reference system II to evaluate $\left(\frac{\partial \mu_1}{\partial \mu_2}\right)_T$. The results from the Gibbs-Duhem equation can be converted to the $\{n_1^{II}\}$ ensemble due to the ensemble invariance of the mole ratio, which leads to

$$\left(\frac{\partial \mu_1}{\partial \mu_2}\right)_T = -\frac{\langle n_2^{II}\rangle_{\{n_1^{II}\}}}{n_1^{II}} \tag{A5}$$

Substituting eq A5 into eq A4 yields the following simplification:

$$-\left(\frac{\partial F}{\partial \mu_2}\right)_T = \langle n_2^*\rangle_{\{n_1\}} - \frac{\langle n_2^{II}\rangle_{\{n_1^{II}\}}}{n_1^{II}} n_1^* \tag{A6}$$

This result is compared to another route: a direct differentiation of eq 4 alongside the relationship equivalent to eq A5, which leads to

$$-\left(\frac{\partial F}{\partial \mu_2}\right)_T = \langle n_2^*\rangle - \frac{\langle n_2^{II}\rangle}{\langle n_1^{II}\rangle}\langle n_1^*\rangle = \Gamma_2^{(1)} \tag{A7}$$

where $\Gamma_2^{(1)}$ is the surface excess (eq 6a). A comparison between eqs A6 and A7 shows that they are two different expressions (in different ensembles) of the same surface excess, $\Gamma_2^{(1)}$. Thus, $\Gamma_2^{(1)}$ is invariant under the ensemble transformation.

B. Calculating the gradient of surface excess via ensemble independence.

Our goal is to relate the gradient of surface excess to sorbate number fluctuations. Since $\Gamma_2^{(1)}$ is invariant, we carry this out efficiently in the $\{T, v, n_e, n_1, \mu_2\}$ ensemble (abbreviated as $\{n_1\}$) before performing statistical variable transformation back to the $\{T, v, n_e, \mu_1, \mu_2\}$ ensemble (abbreviated as $\{\mu_1\}$). First, we differentiate eq A6 in $\{n_1\}$ with respect to μ_2, noting that n_1^* and n_1^{II} are constants, which leads to

$$-kT\left(\frac{\partial^2 F}{\partial \mu_2^2}\right)_T = \langle \delta n_2^* \delta n_2^*\rangle_{\{n_1^*\}} - \frac{n_1^*}{n_1^{II}}\langle \delta n_2^{II} \delta n_2^{II}\rangle_{\{n_1^{II}\}} \tag{B1}$$

where k is the Boltzmann constant. The two number fluctuations in eq B1 can be rewritten in terms of the mole ratio, $C_2 = \frac{n_2}{n_1}$, and its fluctuation, $(\delta C_2)_{\{n_1\}} = (\delta n_2)_{\{n_1\}}/n_1$, as

$$-kT\left(\frac{\partial^2 F}{\partial \mu_2^2}\right)_T = {n_1^*}^2\langle \delta C_2^* \delta C_2^*\rangle_{\{n_1^*\}} - n_1^* n_1^{II}\langle \delta C_2^{II} \delta C_2^{II}\rangle_{\{n_1^{II}\}} \tag{B2a}$$

Since mole ratio fluctuation is invariant under ensemble transformation, the corresponding expression in $\{\mu_1\}$ is expressed as

$$-kT\left(\frac{\partial^2 F}{\partial \mu_2^2}\right)_T = \langle n_1^*\rangle_{\{\mu_1\}}^2\langle \delta C_2^* \delta C_2^*\rangle_{\{\mu_1\}} - \langle n_1^*\rangle_{\{\mu_1\}}\langle n_1^{II}\rangle_{\{\mu_1\}}\langle \delta C_2^{II} \delta C_2^{II}\rangle_{\{\mu_1\}} \tag{B2b}$$

Second, we carry out an ensemble transformation from $\{n_1\}$ to $\{\mu_1\}$. This can be achieved via statistical variable transformation using again the ensemble invariance of the mole ratio, $C_2 = \frac{n_2}{n_1}$, and its deviation from the mean,[1,2] namely,

$$\frac{(n_2)_{\{\mu_1\}} + (\delta n_2)_{\{\mu_1\}}}{(n_1)_{\{\mu_1\}} + (\delta n_1)_{\{\mu_1\}}} = \frac{(n_2)_{\{n_1\}} + (\delta n_2)_{\{n_1\}}}{n_1} \tag{B3}$$

The Maclaurin expansion of eq B3 yields

$$(\delta n_2)_{\{n_1\}} = (\delta n_2 - C_2 \delta n_1)_{\{\mu_1\}} \tag{B4}$$

Substituting eq B4 into eq B1 leads to

$$-kT\left(\frac{\partial^2 F}{\partial \mu_2^2}\right)_T = \langle(\delta n_2^* - C_2^*\delta n_1^*)^2\rangle_{\{\mu_1\}} - \frac{\langle n_1^*\rangle}{\langle n_1^{II}\rangle}\langle(\delta n_2^{II} - C_2^{II}\delta n_1^{II})^2\rangle_{\{\mu_1\}} \quad \text{(B5)}$$

As discussed in the main text, eq B5 is analogous to the cooperative solubilization theory (e.g., eq 46 of Ref [[1]] with the indexes 1 and 2 swapped). Combining eqs A7 and B5, we can write down the fluctuation theory for the gradient of isotherms as

$$\left(\frac{\partial \Gamma_2^{(1)}}{\partial \ln a_2}\right)_T = \langle(\delta n_2^* - C_2^*\delta n_1^*)^2\rangle_{\{\mu_1\}} - \frac{\langle n_1^*\rangle}{\langle n_1^{II}\rangle}\langle(\delta n_2^{II} - C_2^{II}\delta n_1^{II})^2\rangle_{\{\mu_1\}} \quad \text{(B6)}$$

The solid/solution relationship (eq B6) reduces to the solid/gas counterpart when the solvent is dilute ($n_1^*, n_1^{II} \to 0$ and $\delta n_1^*, \delta n_1^* \to 0$), which reduces $\Gamma_2^{(1)} \to \langle n_2^*\rangle$. When the reference phase fluctuation $\langle(\delta n_2^{II})^2\rangle$ is much smaller than at the interface, $\langle(\delta n_2^*)^2\rangle$, we obtain:

$$\left(\frac{\partial \langle n_2^*\rangle}{\partial \ln a_2}\right)_T = \langle(\delta n_2^*)^2\rangle = \langle n_2^*\rangle(N_{22}^* + 1) \quad \text{(B7)}$$

when the sorbate excess number is introduced.[3,4] Thus, our solid/solution sorption theory contains the solid/gas sorption theory as its special case.

C. The "Kirkwood-Buff χ" parameter

Here we clarify the meaning of eq B6 in terms of the Kirkwood-Buff integrals, defined as

$$G_{ij} + \delta_{ij}\frac{v}{\langle n_i\rangle} = \frac{v\langle\delta n_i \delta n_j\rangle}{\langle n_i\rangle\langle n_j\rangle} \quad \text{(C1)}$$

where, for the interface, the ensemble is $\{T, v, n_e, \mu_1, \mu_2\}$ instead of $\{T, v, \mu_e, \mu_1, \mu_2\}$. To do so, let us expand the right-hand side of eq B6 and combine it with eq C1, which yields

$$\langle(\delta n_2 - C_2\delta n_1)^2\rangle_{\{\mu_1\}} = \langle\delta n_2\delta n_2\rangle - 2C_2\langle\delta n_1\delta n_2\rangle + C_2^2\langle\delta n_1\delta n_1\rangle$$
$$= \frac{\langle n_2\rangle^2}{v}\left[G_{22} + G_{11} - 2G_{12} + \frac{v}{\langle n_1\rangle} + \frac{v}{\langle n_2\rangle}\right] \quad \text{(C2)}$$

In the main text, we have introduced the Kirkwood-Buff χ parameter (eq 8b), through which eq C2 can be rewritten as

$$\frac{\langle(\delta n_2 - C\delta n_1)^2\rangle_{\{\mu_1\}}}{\langle n_2\rangle} = C_2\chi + C_2 + 1 \quad \text{(C3)}$$

Using eq C3, eq B6 can be rewritten as

$$\left(\frac{\partial \Gamma_2^{(1)}}{\partial \ln a_2}\right)_T = \langle n_2^*\rangle(C_2^*\chi^* + C_2^* + 1) - \frac{\langle n_1^*\rangle}{\langle n_1^{II}\rangle}\langle n_2^{II}\rangle(C_2^{II}\chi^{II} + C_2^{II} + 1) \quad \text{(C4)}$$

We will show below that eq C4 can be rewritten into the following form

$$\left(\frac{\partial \Gamma_2^{(1)}}{\partial \ln a_2}\right)_T = \langle n_1^*\rangle {C_2^{II}}^2[K^2(\chi^* + 1) - (\chi^{II} + 1)\,] + \Gamma_2^{(1)} \quad \text{(C5a)}$$

where K is the interface/solution sorbate-solvent exchange constant defined as

$$K = \frac{C_2^*}{C_2^{II}} = \frac{\langle n_1^{II}\rangle\langle n_2^*\rangle}{\langle n_1^*\rangle\langle n_2^{II}\rangle} \quad \text{(C5b)}$$

To derive eq C5a, let us start by rewriting eq C4 as

$$\left(\frac{\partial \Gamma_2^{(1)}}{\partial \ln a_2}\right)_T = \left[\langle n_2^*\rangle C_2^*(\chi^* + 1) - \frac{\langle n_1^*\rangle\langle n_2^{II}\rangle}{\langle n_1^{II}\rangle}C_2^{II}(\chi^{II} + 1)\right] + (\langle n_2^*\rangle - C_2^{II}\langle n_1^*\rangle) \quad \text{(C6)}$$

The first term of eq C6 can be simplified using eq C5b as

$$\langle n_2^*\rangle C_2^*(\chi^* + 1) - \frac{\langle n_1^*\rangle\langle n_2^{II}\rangle}{\langle n_1^{II}\rangle}C_2^{II}(\chi^{II} + 1)$$

$$= \langle n_1^* \rangle {C_2^{II}}^2 [K^2(\chi^* + 1) - (\chi^{II} + 1)] \qquad \text{(C7)}$$

which is the first term of eq C5a. The second term of eq C6 is $\Gamma_2^{(1)}$ via eq 6a, which is the second term of eq C5a. Note that the following relationship between $\Gamma_2^{(1)}$ and K,

$$\Gamma_2^{(1)} = \langle n_1^* \rangle C_2^{II}(K - 1) \qquad \text{(C8)}$$

will also be useful.

D. The ABC isotherm for solutions.

Here we derive a statistical thermodynamic isotherm equation as an expansion of the interfacial χ around $a_2 = 0$. To carry this out, we start with the following:

$$\frac{\partial}{\partial a_2} \frac{a_2}{\Gamma_2^{(1)}} = \frac{\Gamma_2^{(1)} - \frac{\partial \Gamma_2^{(1)}}{\partial \ln a_2}}{\left(\Gamma_2^{(1)}\right)^2} \qquad \text{(D1)}$$

Using eqs C5a and C8, eq D1 can be rewritten as

$$\frac{\partial}{\partial a_2} \frac{a_2}{\Gamma_2^{(1)}} = -\frac{K^2(\chi^*+1) - (\chi^{II}+1)}{\langle n_1^* \rangle (K-1)^2} \qquad \text{(D2)}$$

Integrating eq D2 yields

$$\Gamma_2^{(1)} = \frac{a_2}{A - \int_0^{a_2} da_2 \frac{K^2(\chi^*+1) - (\chi^{II}+1)}{\langle n_1^* \rangle (K-1)^2}} \qquad \text{(D3)}$$

where the parameter A has a clear physical interpretation

$$A^{-1} = \left[\frac{\Gamma_2^{(1)}}{a_2}\right]_{a_2 \to 0} = \left[\frac{\langle n_2^* \rangle - \langle n_2^{II} \rangle - C_2^{II}(\langle n_1^* \rangle - \langle n_1^{II} \rangle)}{a_2}\right]_{a_2 \to 0} = c_1^o (G_{s2} - G_{s1})_{a_2 \to 0} \qquad \text{(D4a)}$$

through solvent-surface and sorbate-surface Kirkwood-Buff integrals, defined as

$$G_{s1} = v \frac{\langle n_1^* \rangle - \langle n_1^{II} \rangle}{\langle n_1^{II} \rangle}, \quad G_{s2} = v \frac{\langle n_2^* \rangle - \langle n_2^{II} \rangle}{\langle n_2^{II} \rangle} \qquad \text{(D4b)}$$

where $a_2 \simeq x_2^{II} \simeq \frac{c_2^{II}}{c_1^o}$ at the dilute limit and c_1^o is the bulk molar concentration of solvent. Equation D3 is a general isotherm for adsorption from solution. Here we carry out the activity expansion as

$$\frac{K^2(\chi^*+1) - (\chi^{II}+1)}{\langle n_1^* \rangle (K-1)^2} = B + Ca_2 + \cdots \qquad \text{(D5a)}$$

where

$$B = \left(\frac{K^2(\chi^*+1) - (\chi^{II}+1)}{\langle n_1^* \rangle (K-1)^2}\right)_{a_2 \to 0} \qquad \text{(D5b)}$$

can be interpreted predominantly as the difference of χ between interface and solution (see main text). Combining eqs D3 and D5a yields

$$\Gamma_2^{(1)} = \frac{a_2}{A - Ba_2 - \frac{C}{2}a_2^2} \qquad \text{(D6)}$$

which is the solution-phase generalization of our ABC isotherm for vapor sorption to adsorption form solution.

Our solid/solution parameters are the generalization of our previous theory for solid/gas sorption.[3,4] Neglecting G_{s1} leads to a solid/vapor relationship between G_{s2} and the isotherm.[3,4] When the solvent is absent and $\chi^* \gg 1$ is dominated by G_{22}, eq D5b tends to

$$B \simeq \left(\frac{1}{\langle n_1^* \rangle}\frac{K^2}{K^2}\frac{\langle n_1^* \rangle}{v}G_{22}^*\right)_{a_2 \to 0} = \left(\frac{G_{22}^*}{v}\right)_{a_2 \to 0} \quad \text{(D7)}$$

which is identical to the vapor-phase theory.[3,4] The expression for C involves the three-body correlations that are cumbersome. (Note that χ in eq D5a can be expressed in terms of the ensemble averages of numbers and number-ratio by solving eq 8a for χ. Consequently, the determination of C does not involve any differentiation of v. This was already demonstrated in our recent paper on gas (vapor) sorption.[5])

In the classification of the isotherms, B/A plays a key role. To express this parameter in terms of χ, we must first rewrite eq D4a using in terms of K (eq C5b) as

$$A^{-1} = \left[\frac{\langle n_2^* \rangle - C_2^{II}\langle n_1^* \rangle}{a_2}\right]_{a_2 \to 0} = \left[\langle n_1^* \rangle C_2^{II}\frac{K-1}{a_2}\right]_{a_2 \to 0} = [\langle n_1^* \rangle (K-1)]_{a_2 \to 0} \quad \text{(D8)}$$

We have used $a_2 \simeq x_2^{II} \simeq C_2^{II}$ at $a_2 \to 0$ at the last step of eq D8. Combining eq D5b and D8, we obtain

$$\frac{B}{A} = \left[\frac{K^2(\chi^* + 1) - (\chi^{II} + 1)}{K-1}\right]_{a_2 \to 0} \quad \text{(D9)}$$

which will play a central role in classifying isotherms.

Here we point out that K, representing sorbate-water exchange, is indispensable when working with the χ differences. It is related also to the solvent-surface and sorbate-surface Kirkwood-Buff integrals via eq D4b, as

$$K - 1 = \frac{\langle n_1^{II} \rangle}{\langle n_1^* \rangle}\frac{G_{S2} - G_{S1}}{v} \quad \text{(D10)}$$

E. The ABC isotherm in mole-fraction scale

Let us start with the following equation for x_2, which is analogous to eq D1:

$$\left(\frac{\partial}{\partial x_2^{II}}\frac{x_2^{II}}{\Gamma_2^{(1)}}\right)_T = \frac{\Gamma_2^{(1)} - \frac{\partial \Gamma_2^{(1)}}{\partial \ln x_2^{II}}}{\left(\Gamma_2^{(1)}\right)^2} \quad \text{(E1)}$$

where the differentiation on the right-hand side can be linked to the $\ln a_2$ derivative as

$$\frac{\partial \Gamma_2^{(1)}}{\partial \ln x_2} = \frac{\partial \Gamma_2^{(1)}}{\partial \ln a_2}\frac{\partial \ln a_2}{\partial \ln x_2^{II}} \quad \text{(E2)}$$

By using the following well-known relationship in the Kirkwood-Buff solution theory,

$$\left(\frac{\partial \ln x_2^{II}}{\partial \ln a_2}\right)_{P,T} = 1 + x_2^{II}c_1^{II}(G_{11} + G_{22} - 2G_{12}) = 1 + x_2^{II}\chi^{II} \quad \text{(E3)}$$

and substituting it into eq E1, we obtain

$$\left(\frac{\partial}{\partial x_2^{II}}\frac{x_2^{II}}{\Gamma_2^{(1)}}\right)_T = \frac{1}{1 + x_2^{II}\chi^{II}}\frac{\Gamma_2^{(1)} - \frac{\partial \Gamma_2^{(1)}}{\partial \ln a_2}}{\left(\Gamma_2^{(1)}\right)^2} + \frac{x_2^{II}\chi^{II}}{1 + x_2^{II}\chi^{II}}\frac{1}{\Gamma_2^{(1)}}$$

$$= \frac{1}{1 + x_2^{II}\chi^{II}}\frac{\partial}{\partial a_2}\frac{a_2}{\Gamma_2^{(1)}} + \frac{x_2^{II}\chi^{II}}{1 + x_2^{II}\chi^{II}}\frac{1}{\Gamma_2^{(1)}} \quad \text{(E4)}$$

Using eq D2, eq E4 can be expressed as

$$\left(\frac{\partial}{\partial x_2^{II}}\frac{x_2^{II}}{\Gamma_2^{(1)}}\right)_T = -\frac{1}{1 + x_2^{II}\chi^{II}}\frac{1}{\langle n_1^* \rangle}\frac{K^2(\chi^* + 1) - (\chi^{II} + 1)}{(K-1)^2} + \frac{x_2^{II}\chi^{II}}{1 + x_2^{II}\chi^{II}}\frac{1}{\Gamma_2^{(1)}} \quad \text{(E5)}$$

Here we introduce the x_2-expansion in a manner analogous to eqs D2 and D5a, as

$$\frac{1}{1+x_2^{II}\chi^{II}}\frac{1}{\langle n_1^*\rangle}\frac{K^2(\chi^*+1)-(\chi^{II}+1)}{(K-1)^2}-\frac{x_2^{II}\chi^{II}}{1+x_2^{II}\chi^{II}}\frac{1}{\Gamma_2^{(1)}}=B_x+C_x x_2^{II} \quad \text{(E6)}$$

through which eq E5 can be integrated to yield

$$\Gamma_2^{(1)}=\frac{x_2^{II}}{A_x-B_x x_2^{II}-\frac{C_x}{2}x_2^{II^2}} \quad \text{(E7)}$$

where the integration constant, A_x, can be shown to be identical to A in the a_2-based isotherm, because

$$A_x^{-1}=\left(\frac{\Gamma_2^{(1)}}{x_2}\right)_{x_2^{II}\to 0}=\left(\frac{\Gamma_2^{(1)}}{a_2}\right)_{a_2\to 0}=A^{-1} \quad \text{(E8)}$$

due to the dilute-ideal condition. At the same limit,

$$B_x=\left(\frac{1}{1+x_2^{II}\chi^{II}}\frac{1}{\langle n_1^*\rangle}\frac{K^2(\chi^*+1)-(\chi^{II}+1)}{(K-1)^2}-\frac{x_2^{II}\chi^{II}}{1+x_2^{II}\chi^{II}}\frac{1}{\Gamma_2^{(1)}}\right)_{x_2^{II}\to 0}$$

$$=\left(\frac{1}{\langle n_1^*\rangle}\frac{K^2(\chi^*+1)-(\chi^{II}+1)}{(K-1)^2}\right)_{x_2^{II}\to 0}=B \quad \text{(E9)}$$

and the parameter C_x would be significantly more complex than C. Thus, we rewrite eq E7 as

$$\Gamma_2^{(1)}=\frac{x_2^{II}}{A-Bx_2^{II}-\frac{C_x}{2}x_2^{II^2}} \quad \text{(E10)}$$

by appreciating that A and B are the same as in the activity-based isotherm.

F. The cubic isotherm.

The ABC isotherm faces difficulties when $\Gamma_2^{(1)}$ becomes negative. Hence a different isotherm equation is needed for fitting. To this end, let us start by rewriting eq D2 as

$$\frac{\partial}{\partial a_2}\frac{\Gamma_2^{(1)}}{a_2}=\frac{\Gamma_2^{(1)^2}}{a_2^2}\frac{1}{\langle n_1^*\rangle}\frac{K^2(\chi^*+1)-(\chi^{II}+1)}{(K-1)^2}=\left(\frac{C_2^{II}}{a_2}\right)^2\langle n_1^*\rangle[K^2(\chi^*+1)-(\chi^{II}+1)] \quad \text{(F1)}$$

In a parallel manner to eq D5a, we introduce the sorbate activity expansion, as

$$\left(\frac{C_2^{II}}{a_2}\right)^2\langle n_1^*\rangle[K^2(\chi^*+1)-(\chi^{II}+1)]=B'+C'a_2 \quad \text{(F2)}$$

Integrating eq F1 with eq F2 yields the following polynomial isotherm:

$$\Gamma_2^{(1)}=A'a_2+B'a_2^2+\frac{C'}{2}a_2^3 \quad \text{(F3a)}$$

where A' was introduced upon integration. Similarly, the x_2^{II} representation can be derived as

$$\Gamma_2^{(1)}=A_x'x_2^{II}+B_x'x_2^{II^2}+\frac{C_x'}{2}x_2^{II^3} \quad \text{(F3b)}$$

Now we show that the parameters A_x', B_x', and C_x', can be expressed in terms of A, B, and C_x of the ABC isotherm. To do so, all we need to do is to compare eq F3b with the Maclaurin expansion of the ABC isotherm (eq 15) as

$$\Gamma_2^{(1)}=\frac{1}{A}x_2^{II}+\frac{B}{A^2}x_2^{II^2}+\frac{1}{2}\left(\frac{C_x}{A^2}+\frac{2B^2}{A^3}\right)x_2^{II^3} \quad \text{(F4)}$$

Comparing the parameters of eqs F3b and F4 yields the following relationship between the cubic and ABC isotherms:

$$A_x'=\frac{1}{A},\ B_x'=\frac{B}{A^2},\ C_x'=\frac{C_x}{A^2}+\frac{2B^2}{A^3} \quad \text{(F5)}$$

Thus, the same set of parameters (A,B,C_x) can be determined both from the ABC and cubic isotherms.

References

(1) Shimizu, S.; Matubayasi, N. Intensive Nature of Fluctuations: Reconceptualizing Kirkwood-Buff Theory via Elementary Algebra. *J. Mol. Liq.* **2020**, *318*, 114225. https://doi.org/10.1016/j.molliq.2020.114225.

(2) Shimizu, S.; Matubayasi, N. Ensemble Transformation in the Fluctuation Theory. *Physica A* **2022**, *585*, 126430. https://doi.org/10.1016/J.PHYSA.2021.126430.

(3) Shimizu, S.; Matubayasi, N. Fluctuation Adsorption Theory: Quantifying Adsorbate-Adsorbate Interaction and Interfacial Phase Transition from an Isotherm. *Phys. Chem. Chem. Phys.* **2020**, *22*, 28304–28316. https://doi.org/10.1039/D0CP05122E.

(4) Shimizu, S.; Matubayasi, N. Sorption: A Statistical Thermodynamic Fluctuation Theory. *Langmuir* **2021**, *37*, 7380–7391. https://doi.org/10.1021/acs.langmuir.1c00742.

(5) Shimizu, S.; Matubayasi, N. Understanding Sorption Mechanisms Directly from Isotherms. *Langmuir* **2023**, *39* (17), 6113–6125. https://doi.org/10.1021/acs.langmuir.3c00256.
